# Study on the Influence and Performance of Nano SiO_2_ on Solid Waste Grouting Material

**DOI:** 10.3390/ma18174110

**Published:** 2025-09-01

**Authors:** Huifang Zhang, Lei Wang, Jie Chen, Haiyang Chen, Wei Wu, Jinzhu Li, Henan Lu, Dongxiao Hu, Hongliang Huang

**Affiliations:** 1 College of Civil Engineering, Hebei University of Architecture, Zhangjiakou 075000, China; zhfdayanjing09@163.com (H.Z.); 18406463229@163.com (L.W.); 18816408823@163.com (J.C.); huanghong.5055@163.com (H.H.); 2 Underground Engineering Technology Innovation Center of Hebei Province, Zhangjiakou 075000, China; 3 Qingshui River Administration Center, Zhangjiakou 075000, China; w18406463229@126.com; 4 Construction Engineering Quality Service Center, Zhangjiakou 075000, China; c18816408823@126.com; 5 Zhangjiakou Jianfa Water Affairs Group Co., Ltd., Zhangjiakou 075000, China; g1771623330@163.com; 6 School of Materials Science and Engineering, University of Science and Technology Beijing, Beijing 100083, China; quanquanquan686@163.com

**Keywords:** sleeve grouting material, nano-SiO_2_, silica fume, half grouted sleeve, microscopic analysis, mechanism analysis

## Abstract

As a key connection technology in prefabricated buildings, offshore wind power, and bridge engineering, the performance and environmental sustainability of grouted sleeve connections are essential for the long-term development of civil infrastructure. To address the environmental burden of conventional high-strength cement-based grouts, an eco-friendly sleeve grouting material incorporating industrial solid waste was developed. In this study, silica fume (15%) and fly ash (5%) were employed as supplementary cementitious materials, while nanosilica (NS) was introduced to enhance the material properties. Mechanical testing, microstructural characterization, and half-grouted sleeve uniaxial tensile tests were conducted to systematically evaluate the effect of NS content on grout performance. Results indicate that the incorporation of NS significantly accelerates the hydration of silica fume and fly ash. At an optimal dosage of 0.4%, the 28-day compressive strength reached 105.5 MPa, representing a 37.9% increase compared with the control group without NS. In sleeve tensile tests, specimens with NS exhibited reinforcement necking failure, and the load–displacement response closely aligned with the stress–strain behavior of the reinforcement. A linear relationship was observed between sleeve wall strain and reinforcement stress, confirming the cooperative load-bearing behavior between the grout and the sleeve. These findings provide theoretical guidance and technical support for developing high-strength, low-impact grouting materials suitable for sustainable engineering applications.

## 1. Introduction

With China’s economic transition from high-speed growth to high-quality development, the industrial structure has undergone continuous upgrading and optimization, and all sectors are increasingly committed to efficiency, energy conservation, and green development [1,2]. As a key trend in the construction industry, prefabricated buildings have exhibited robust growth in recent years. Driven by supportive policies and market demand, the industrial capacity of the prefabricated construction sector has expanded rapidly [3].

Grouted sleeves, as prefabricated components for rebar connections, are primarily used in precast concrete structures. The grouting material is injected between the rebar and the inner wall of the sleeve to ensure the structural integrity of the connection. It is typically composed of cement, standard sand, chemical admixtures, and water [4,5]. China’s cross-sea bridge projects, such as the Hong Kong–Zhuhai–Macao Bridge and the Shenzhen–Zhongshan Link, have adopted the innovative construction method of “factory prefabrication + on-site assembly” in several key structural sections. However, cement production is highly energy-intensive and contributes significantly to CO_2_ emissions, while the excessive exploitation of standard sand poses threats to resource sustainability and environmental compliance with current policies [6,7]. Although various researchers have explored the use of solid waste materials and optimized their mix proportions to achieve favorable workability and compressive strength, meeting the stringent performance requirements of sleeve grouts remains a challenge [8,9,10]. Therefore, nanosilica (NS) has been introduced as a performance-enhancing additive to improve the properties of solid waste–based grouting materials.

In the field of cement-based composites, the application of nanomaterials has attracted increasing attention in recent years due to their potential to enhance the performance of these materials by modifying the microstructure at the nanoscale [11,12]. Xiaoman Xie et al. [13] studied the application of nanomaterial-modified microcapsules (silica, graphene oxide) in cement self-healing and found that it can reduce crack width by 48% and achieve surface healing within 4 h, thereby improving the durability and mechanical properties of cement. Dan Wang et al. [14] prepared nano-silica (S-20) using the sol–gel method, which resulted in a 28.8% reduction in water absorption and a 57.5% reduction in chloride ion permeability, and promoted the formation of a more compact microstructure in cement-based materials. Chunlei Yao et al. [15] found that mixing 3% nano-silica (NS) with 10% fly ash (FA) can increase the 28-day compressive strength by 18.52 MPa, improve the expansion rate by 12.9%, and increase methane extraction efficiency by 3.5% in engineering applications. Huang Lingzhi et al. [16] reported that the incorporation of nanosilica (NS) induces a nucleation effect and chemically reacts with calcium hydroxide crystals, resulting in the formation of coral-like and spherical hydration products such as calcium silicate hydrate (C-S-H) gel and calcium ferrite hydrate (C-F-H) gel within the concrete matrix. The highly reactive nucleation sites facilitate the aggregation of hydration gels on their surfaces, thereby reducing the amount of Ca(OH)_2_ and filling voids to form a denser and more compact microstructure. Moreover, NS has been shown to significantly improve the mechanical properties, durability, and microstructure of cement-based materials. Numerous studies have demonstrated that even a small dosage of nanomaterials can substantially enhance both the static and dynamic mechanical properties of concrete [17,18]. Research by Hongyin Hu et al. [19] shows that the incorporation of nano-zinc oxide and nano-zirconium dioxide significantly improves the compressive strength of cement-based materials (lower limit increased by 88.17%, upper limit increased by 15.14%) and reduces water absorption. Among these, nano-zirconium dioxide can mitigate quality loss caused by self-shrinkage and drying shrinkage. Penggang Wang et al. [20] combined modified ethyl cellulose microcapsules with nano-silica (NS) to achieve a compressive strength recovery rate of 151.2% in concrete and optimized the pore and crack healing effect at an NS content of 6%. In research on nanomodified cement-based materials, Zhenjun Wang et al. [21] found that polycarboxylic acid superplasticizers (PCE) synergistically interact with ultrasonic treatment to significantly improve the dispersion of nano-silica (NS). Experiments showed that a 1.0% PCE concentration combined with one hour of ultrasonic treatment reduced the porosity of the cement paste to 3.07% and increased the compressive strength at 28 days by 21.6%, revealing the synergistic enhancement mechanism between NS dispersion and the mechanical properties and microstructure of cement-based materials. Wei Chao [22], in developing high-performance sleeve grouting materials with NS, emphasized that excessive NS content can severely impair the grout’s fluidity. Zheng, Y. et al. [23] summarized recent domestic and international research indicating that when modified recycled cement is blended with NS, its pozzolanic activity and nucleation effect shorten setting time, increase water absorption, and enhance the capillary absorption coefficient. However, an excessive amount of NS may reduce strength, with optimal performance achieved at an NS content of 2%.

According to existing studies, most investigations on the dosage of nano-silica (NS) have focused on the range of 2–8%, with emphasis placed on improving the performance of grouting materials for sleeves, while comparatively less attention has been given to the sustainability of construction materials. To meet the requirements for mechanical properties and fluidity of grouting materials specified in the Technical Specification for Grouted Connection of Rebar Sleeve [24] (JGJ 355-2015), this study employed a controlled variable approach. On the basis of incorporating solid waste materials such as silica fume, fly ash, quartz sand, and iron tailings sand, the NS content was controlled within the range of 0.2–1% to optimize the grouting material and analyze its influencing mechanisms. Subsequently, pullout tests of reinforcing bars were conducted using semi-grouted sleeves with diameters of 16 mm and 18 mm to evaluate the actual compatibility of the grouting material. The findings are expected to provide a useful reference for integrating novel nanomaterials with solid waste materials in the field of prefabricated construction.

## 2. Materials and Methods

### 2.1. Materials

#### 2.1.1. Cement

P·O 52.5 ordinary Portland cement was used in this study. In the preliminary tests, the mechanical properties, setting time, and specific surface area of the cement were measured, and the fundamental properties are summarized in Table 1.

#### 2.1.2. Silica Fume, Fly Ash, and NS

The silica fume used in this study contained more than 90% SiO_2_, with particle sizes below 1 µm and an average particle diameter of approximately 0.2 µm. Most of the particles exhibited an almost perfectly spherical shape and relatively smooth surfaces. The particle size distribution is shown in Table 2. The fly ash used in this study was classified as Grade I, featuring a relatively large specific surface area. The particles were generally regular in shape but had rough and uneven surfaces, contributing to high adsorption activity. According to the data provided by the material manufacturer, the fundamental properties are presented in Table 2 and Table 3.

The nanosilica (NS) used in this study appeared as white powders, with commonly available particle sizes of 10 nm, 20 nm, 50 nm, 100 nm, and 200 nm. In this experiment, spherical NS with a particle size of 20 nm were selected. The fundamental performance indicators are presented in Table 4.

#### 2.1.3. Microstructure of Cementitious Materials

**Figure 1:**
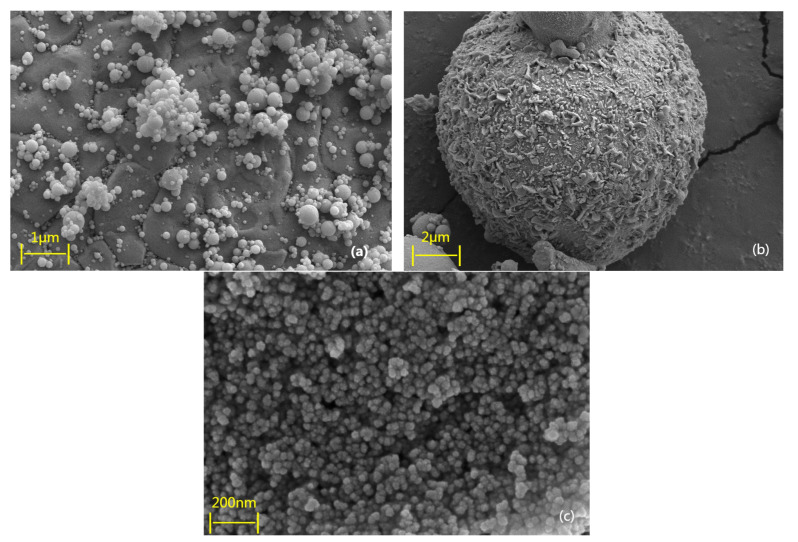
Microstructure of cementitious materials: (**a**) silica fume; (**b**) fly ash; (**c**) NS.

Figure 1a–c presents the microstructural morphologies of silica fume, fly ash, and NS, respectively. The silica fume particles predominantly exhibit a nearly perfect spherical shape with smooth surfaces. This morphology results from the rapid condensation of a high-temperature gas mixture of silicon dioxide and silicon reacting vigorously with oxygen, forming amorphous, non-crystalline spherical particles under the influence of surface tension. Such a shape imparts a “ball-bearing” effect in concrete, thereby enhancing its workability and flowability. Most fly ash particles appear as solid glassy spheres of various sizes, typically darker in color, with rough, uneven surfaces and a clearly visible porous structure. NS particles are mostly spherical with relatively smooth surfaces and exhibit small, uniform particle sizes. Some NS particles are present as agglomerates, consisting of clusters of multiple spherical particles adhered together.

#### 2.1.4. Fine Aggregate

The fine aggregates used in this study consisted of quartz sand and iron tailings sand. The iron tailings sand was obtained from the waste residues of the Xuan Hua Iron Mine in Zhangjiakou, China. The corresponding performance indicators are provided in Table 5.

#### 2.1.5. Half Grouted Sleeve

**Figure 2:**
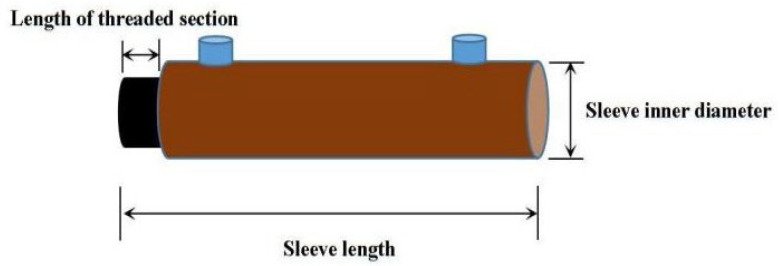
Schematic diagram of semi-grouted sleeve.

The reinforcing bars used in this study were HRB400 hot-rolled ribbed steel bars (Manufactured by Hebei Jingye Company, located in Shijiazhuang City, Hebei Province, China). The yield strength of the bars exceeded 350 MPa, with a measured value of approximately 440 MPa. The tensile strength was greater than 600 MPa, with a measured tensile strength of about 660 MPa. The elongation after fracture was approximately 12%, meeting the requirements of the Technical Specification for Grouted Sleeve Used for Rebar Splicing [25] (JG/T 398-2019). The diameters of the steel bars were 16 mm and 18 mm, respectively, and the actual anchorage length exceeded eight times the bar diameter. Ductile cast iron semi-grouted sleeves, matched to the corresponding bar sizes, were used. The mechanical properties of the reinforcing bars are listed in Table 6, the physical parameters of the grouted sleeve specimens are provided in Table 7, and a schematic diagram of the semi-grouted sleeve is shown in Figure 2.

### 2.2. Mix Design of Solid Waste Composite Materials with NS Admixture

The grout was prepared by partially replacing cement with silica fume (15%) and fly ash (5%), while using iron tailings sand (20%) and quartz sand (80%) as fine aggregates. Nanosilica (NS) was added at five different dosages—0.2%, 0.4%, 0.6%, 0.8%, and 1.0%—to investigate its influence on the performance of sleeve grout incorporating solid waste. The group without NS addition was designated as the control group (G2). The detailed mix proportions are listed in Table 8.

### 2.3. Mechanical Property Test

According to the Test Methods for Strength of Cement Mortar (ISO) [26] (GB/T 17671-2021), specimens with dimensions of 40 mm × 40 mm × 160 mm were prepared. After curing in a standard curing room for 24 h, the specimens were demolded and subjected to water curing until the designated testing age, at which point flexural and compressive strength tests were conducted. The standards referenced for flowability and strength evaluations included the Technical Specification for Application of Grouted Sleeve Connections for Reinforcement (JGJ 355-2015), the Flowability Test Method for Cement Mortar [27] (GB/T 2419-2005), and the Technical Specification for Application of Cement-Based Grouting Materials [28] (GB/T 50448-2015).

### 2.4. Microscopic Test

X-ray diffraction (XRD) analysis was performed using a Miniflex 600 X-ray diffractometer manufactured by Rigaku Corporation (Tokyo, Japan) with a scanning range of 10° to 80°, a step size of 0.02°, and a dwell time of 0.5 s per step. Scanning electron microscopy (SEM) imaging was conducted using a MERLIN Compact scanning electron microscope manufactured by Carl Zeiss AG (Oberkochen, Germany) at an accelerating voltage of 3 kV and a working distance of 7.9 to 8.9 mm.

### 2.5. Preparation of Grouting Sleeve and Joint Type Inspection

#### 2.5.1. Preparation of Grouting Sleeve

**Figure 3:**
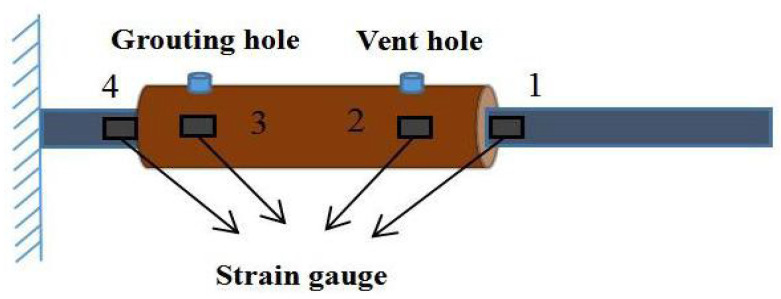
Adhesive of strain gauges on the surface of the sleeve.

**Figure 4:**
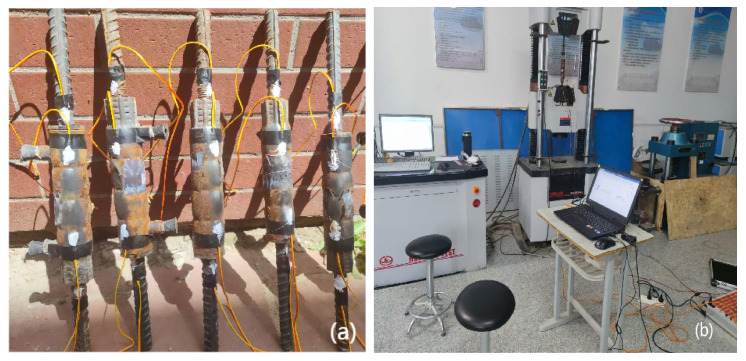
(**a**) Half-grouted sleeve; (**b**) Loading device.

According to the Technical Specification for Application of Grouted Sleeve Connections for Reinforcement [24] (JGJ 355-2015), a total of 24 grouted sleeves were prepared based on six grout mix designs, with two sleeves per group and sleeve diameters of 16 mm and 18 mm. After preparing the grout according to the specified proportions, grouting tests were performed using a handheld grouting injector. The specimens were cured under standard conditions until reaching the designated age of 28 days. Strain gauges were attached to the surface of the grouted sleeves and at both ends of the sleeve-to-rebar connections after surface grinding. The stress–strain behavior of the grouted sleeves was then analyzed using the DHDAS dynamic signal acquisition and analysis system (Jiangsu Donghua Vibration Test Technology Co., Ltd., Jingjiang, China). The locations of the strain gauge attachments are shown in Figure 3, the semi-grouted sleeves with attached gauges are illustrated in Figure 4a, and the loading setup is presented in Figure 4b.

#### 2.5.2. Grout Coupling Joint Type Test

During the joint type testing, according to the Technical Specification for Application of Grouted Sleeve Connections for Reinforcement (JGJ 355-2015), uniaxial tensile tests primarily evaluate deformation and strength. The deformation assessment encompasses residual deformation and total elongation at maximum load, with specific criteria detailed in Table 9. The strength evaluation focuses on the ultimate load-bearing capacity of the joint. The tensile strength of the grouted sleeve connection should be equal to or greater than the standard tensile strength of the connected reinforcing bars, and failure is required to occur in the steel bar outside the joint region.

Uniaxial tensile tests on the reinforcing bars were conducted using a microcomputer-controlled universal testing machine. The loading procedure was as follows: initially, the load was applied from zero up to 60% of the standard yield strength of the test rebar. The load was then removed until the internal force in the rebar returned to zero, at which point the residual deformation was measured and recorded. After this, loading was resumed from zero and continued until the rebar reached its maximum tensile load, where the ultimate tensile strength was recorded. Loading was further applied until specimen failure, and the total elongation at maximum stress was determined through measurement.

1. Residual deformation

**Figure 5:**
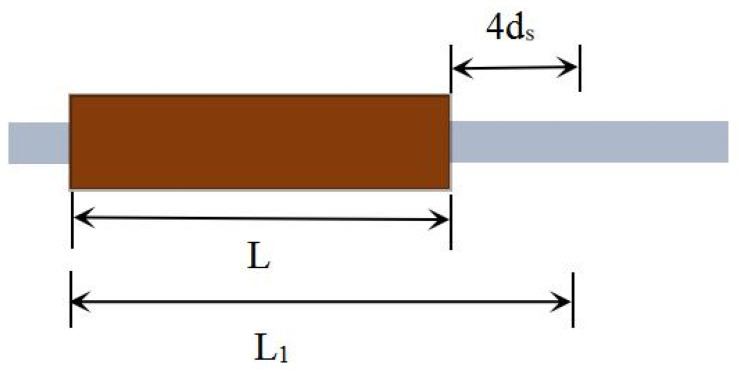
Schematic diagram of residual deformation measurement.

In the uniaxial tensile test, the residual deformation was determined by averaging the instrument readings. The gauge length for deformation measurement was defined as L_1_ = L + 4d_s_, where L1 is the deformation measurement gauge length, L is the length of the grouted sleeve, and ds is the nominal diameter of the reinforcing bar (Figure 5).

2. Total elongation at maximum force

**Figure 6:**
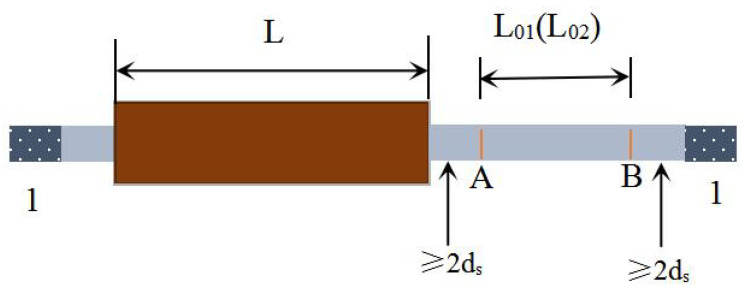
Schematic diagram of the maximum force total.

Before conducting the loading test, fine lines were marked on the surfaces of the reinforcing bars at both ends of the grouted sleeve specimen to designate points A and B, clearly defining the measurement gauge length L_01_. According to the specifications, L_01_ must exceed 100 mm to ensure measurement accuracy. Furthermore, to obtain precise measurements, the gauge length should be measured using high-precision instruments with a minimum scale resolution of 0.1 mm or better. After completion of the uniaxial tensile loading test, the gauge length at both ends was remeasured and denoted as L_02_. The total elongation under maximum load can then be calculated based on the difference between L_02_ and L_01_, enabling an evaluation of the specimen’s deformation capacity during tensile loading (Figure 6). Region 1 corresponds to the rebar anchorage zone, with the calculation formula provided in Equation (1).

(1) $$A_{sgt}=\left[\frac{L_{02}-L_{01}}{L_{01}}+\frac{f_{mst}^{0}}{E}\right]\times 100\%$$

Annotation: L_01_ represents the distance length between A and B before loading, with the unit being mm; L_02_ represents the distance length between A and B after loading, with the unit being mm; $f_{mst}^{0}$ represents the stress of the steel bar when the maximum force is reached, with the unit being N/m^2^; E represents the theoretical elastic modulus of the steel bar, with the unit being N/m^2^.

## 3. Results

### 3.1. The Effect of NS on the Compressive Strength and Flowability of Grout

**Figure 7:**
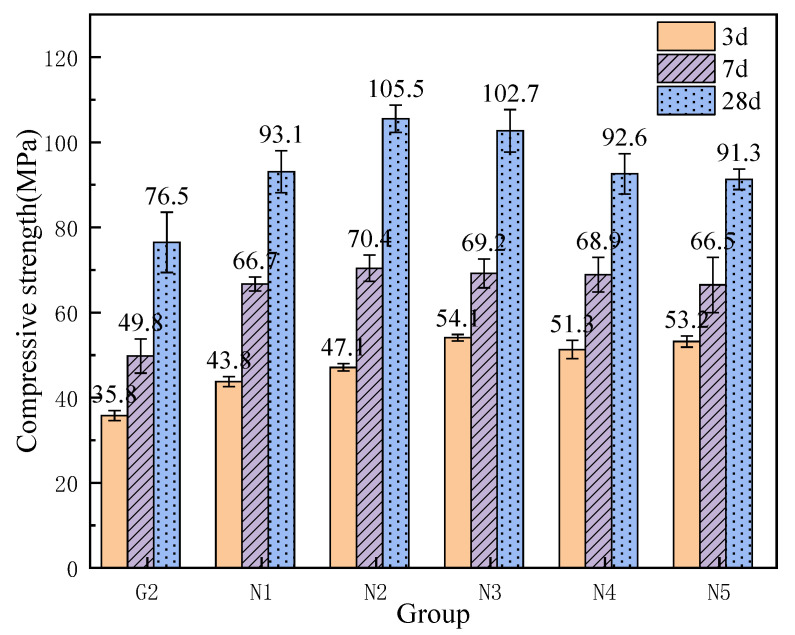
Compressive strength of NS grouting material.

**Figure 8:**
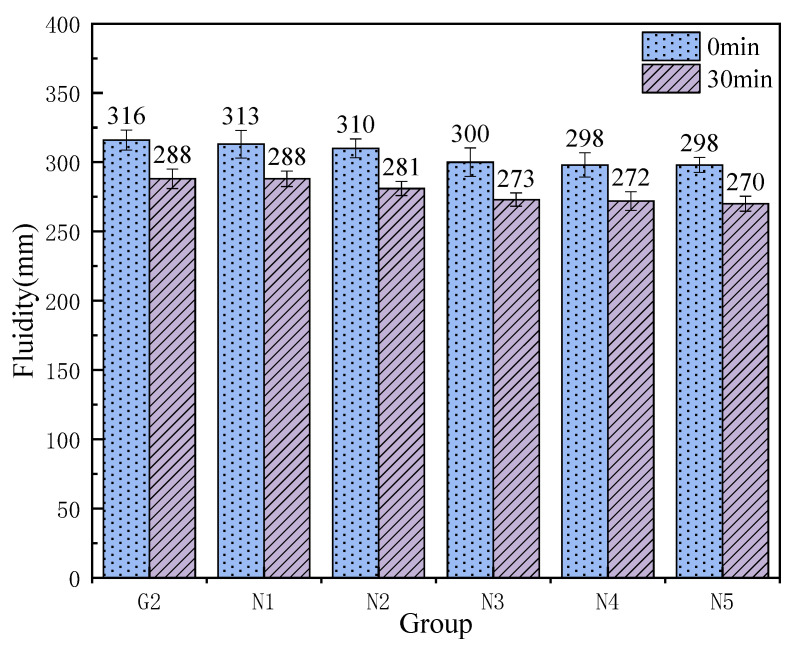
Flowability of NS grouting material.

As shown in Figure 7, the incorporation of nanosilica (NS) effectively enhances the 3-day compressive strength of the grout, exhibiting a trend of initial increase followed by a decline. The strength improvement in groups N1 and N2 is less pronounced compared to groups N3 through N5. Notably, group N3, with an NS content of 0.6%, achieved the highest 3-day compressive strength of 54.1 MPa, approximately 18.3 MPa higher than the control group G2. This suggests that a relatively higher NS dosage significantly promotes early strength development of the grout. The 7-day compressive strength shows minimal variation among groups N1 to N5, indicating that NS dosage has a limited influence on the 7-day strength. However, compared to group G2, NS addition notably improves the 7-day compressive strength, with group N2 (0.4% NS) reaching a maximum value of 70.4 MPa. Furthermore, Figure 8 clearly shows that the 28-day compressive strength follows a pattern of increasing initially and then decreasing. Both groups N2 and N3 exhibit compressive strengths exceeding 100 MPa, at 105.5 MPa and 102.7 MPa, respectively. The optimal group N2 surpasses the control group G2 by approximately 29 MPa, representing an improvement of about 37.9%. Groups N1, N4, and N5 also maintain compressive strengths around 92 MPa, indicating that NS incorporation effectively enhances the 28-day compressive strength of the grout, with 0.4% identified as the relatively optimal dosage.

As shown in Figure 8, with increasing nanosilica (NS) content, both the initial flowability and the flowability after 30 min show a continuous decline compared to the control group G2, with values consistently lower than those of G2. For groups N3, N4, and N5, the initial flowability fluctuates around 300 mm, with the lowest value of 298 mm observed in group N4. Nonetheless, the initial and 30-min flowability values of all groups meet the grout specification standards. Considering the grout’s mechanical properties comprehensively, although the flowability values of groups N1 and N2 are lower than those of group G2, they still largely comply with the specification requirements. Furthermore, the 7-day and 28-day compressive strengths of group N2 exceed those of group N1. Therefore, group N2, with an NS content of 0.4%, is identified as the optimal mix proportion.

### 3.2. Phase Analysis

**Figure 9:**
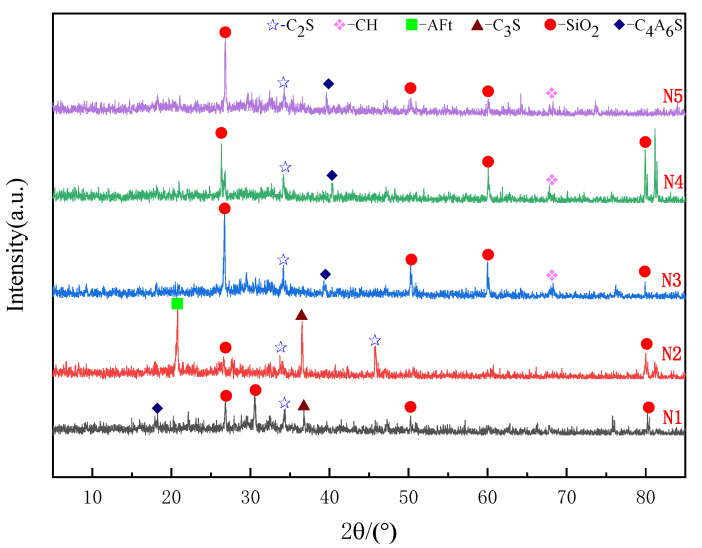
N1–N5 XRD spectra.

XRD analysis results, as shown in Figure 9, indicate that the primary hydration products of the cement-based grout are dicalcium silicate (C_2_S), tricalcium silicate (C_3_S), ettringite (AFt), calcium hydroxide (CH), and calcium sulfoaluminate (C_4_A_6_S). A broad peak observed between 20° and 45° corresponds to the formation of calcium silicate hydrate (C-S-H) gel and ettringite crystals during the hydration reaction. The formation of these hydration products contributes to the enhancement in the specimen’s flexural and compressive strengths.

As shown in Figure 9, compared with other groups, the N2 group generated more hydration products such as C2S, C3S, and AFt, with higher peak values for the hydration products. Among them, the peak value of the calcium aluminate crystal was obvious, indicating that when the NS content was 0.4%, the calcium aluminate crystal was complete and had good crystallinity.

**Figure 10:**
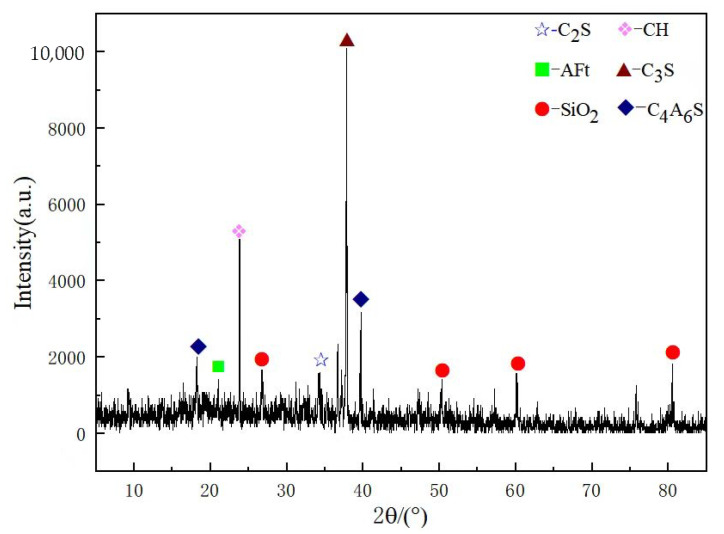
G2 XRD spectra.

**Figure 11:**
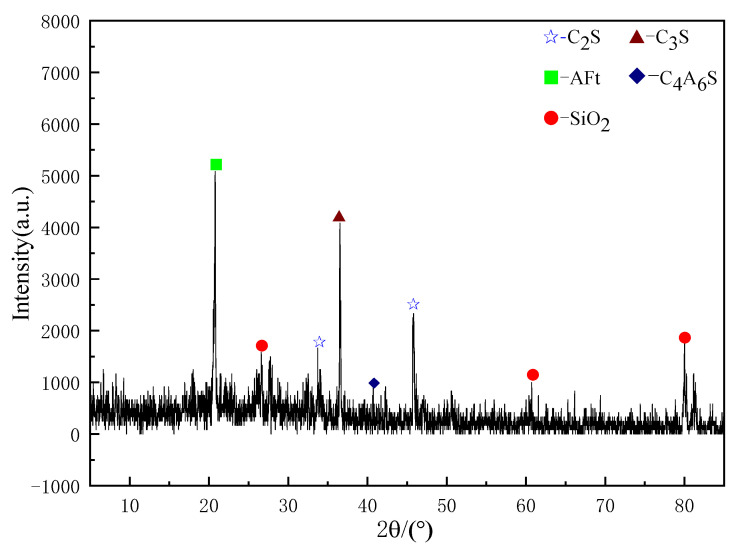
N2 XRD spectra.

Plot Figure 10 and Figure 11 using XRD data from the N2 and G2 groups separately. Observing Figure 10, it can be seen that the G2 group has a high peak for CH, but with the addition of NS, CH is consumed, and the peaks of hydration products such as AFt show a significant increase (as shown in Figure 11). This is due to the nucleation effect of NS, which can adsorb CH produced by cement hydration, promote the forward progression of the hydration reaction while reacting with CH, and further generate AFt and C-S-H gel. This has a significant impact on the strength and durability of the cement stone, manifested in the material’s macro perspective with relatively higher flexural and compressive strengths.

### 3.3. Micro Mechanism Analysis

**Figure 12:**
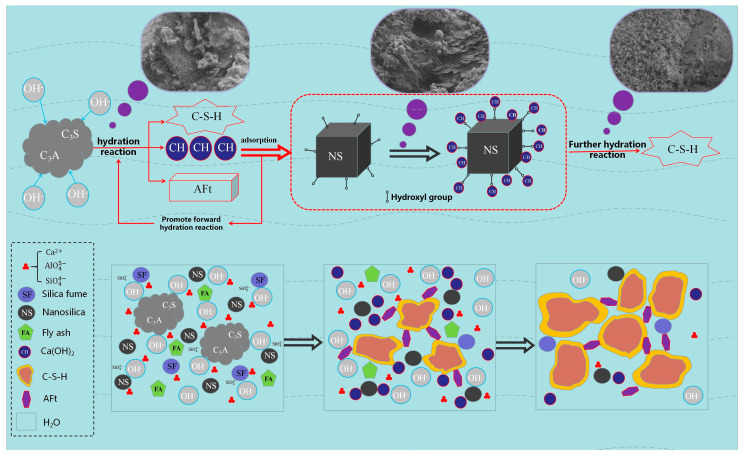
Mechanism diagram of NS promoting the hydration reaction.

**Figure 13:**
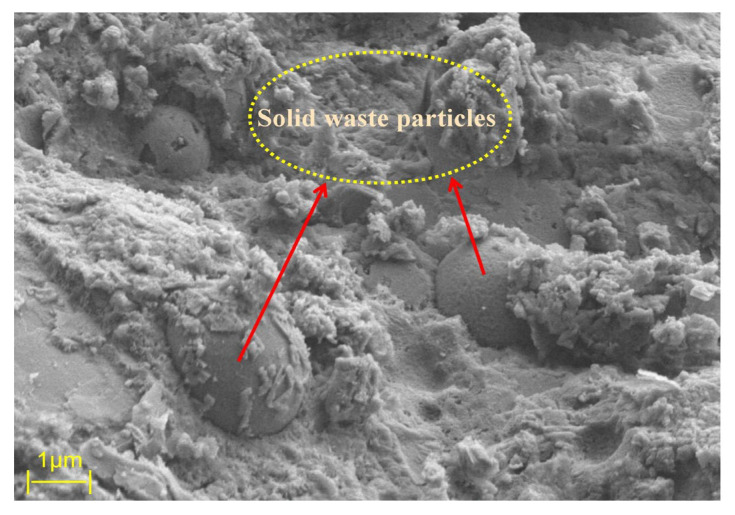
SEM photo of G2.

**Figure 14:**
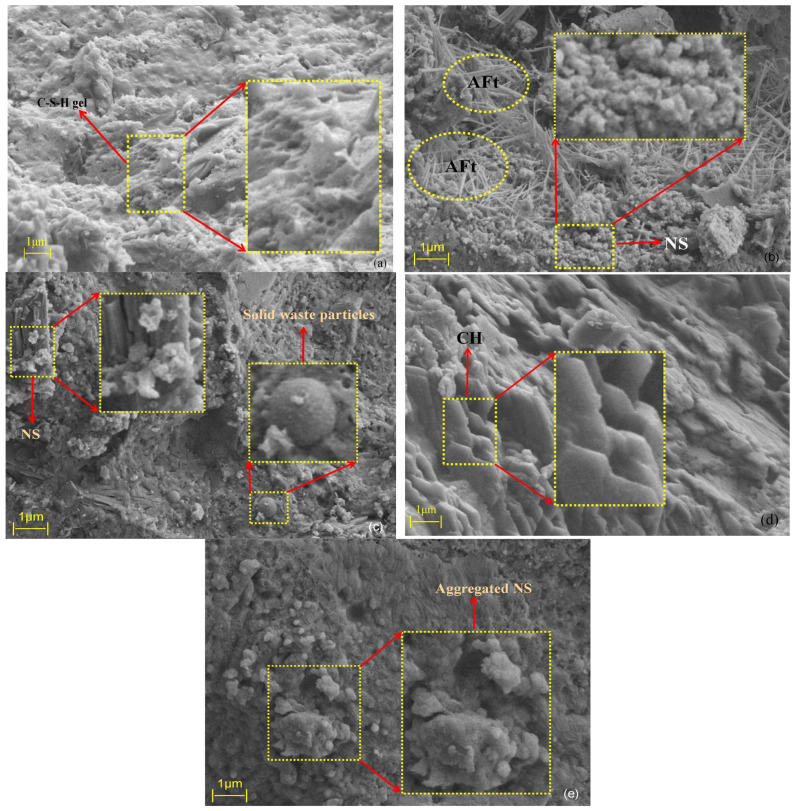
SEM photo of N1–N5: (**a**) N1; (**b**) N2; (**c**) N3; (**d**) N4; (**e**) N5.

From a microstructural perspective, the primary source of strength in grout materials is the formation of calcium silicate hydrate (C-S-H) gel, which is generated through the hydration reaction between silicate ions and calcium ions in the cement. The main role of the C-S-H gel is to fill pores, thereby improving the density and mechanical strength of the material. Figure 12 illustrates the mechanism by which nanosilica (NS) promotes the hydration reaction. Figure 13 shows the SEM image of the control group G2, while Figure 14a–e display the SEM images of groups N1 through N5, respectively.

Based on the SEM images in Figure 13 and Figure 14, it can be observed that the hydration of the cement-based grout produces rod-like and needle-like ettringite (AFt) crystals, reticulated C-S-H gel, plate-like calcium hydroxide (CH) crystals, as well as partially reacted fly ash and silica fume particles. CH is a typical hydration product of cement; however, excessive CH can consume free Ca^2+^ ions in the paste, thereby inhibiting the formation of C-S-H gel. This limitation can lead to a decrease in grout strength, adversely affecting the overall mechanical performance to some extent.

With the incorporation of nanosilica (NS), the hydration behavior of the cement-based grout is significantly affected. Due to abundant hydroxyl groups on the NS surface, which are polar and highly hydrophilic, NS strongly adsorbs calcium hydroxide (CH) produced during cement hydration. This adsorption reduces the concentration of CH in the pore solution, disrupting the hydration equilibrium and promoting further hydration of cement minerals. Moreover, NS reacts with CH in a secondary pozzolanic reaction, generating additional C-S-H gel. These newly formed C-S-H gels fill the pores in the cement matrix, further enhancing the grout’s density and mechanical strength. A schematic diagram of the hydration mechanism is shown in Figure 11.

Comparing different dosages of nanosilica (NS), Figure 14a shows no obvious unreacted solid waste particles, with strong overall material connectivity. Clustered C-S-H gel is well bonded to the cement matrix, and abundant reticulated C-S-H gel interconnects with needle-like AFt crystals. In Figure 14b, numerous fibrous C-S-H gel and needle-rod-shaped AFt crystals clearly bond the surrounding material, forming a structure akin to reinforced concrete. Crystalline AFt fills pores at gel interfaces within the matrix, improving internal pore structure and resulting in higher strength in the hardened cement-based grout. Figure 14c reveals many fine NS particles adsorbed on the surfaces of silica fume, fly ash, and CH, accelerating the hydration process; however, some unreacted spherical silica fume or fly ash particles are still visible. Overall, group N2 exhibits slightly higher compressive strength than group N3 at the macroscopic level. Figure 14d clearly displays hexagonal plate-like or layered CH crystals with attached NS particles, but excessive CH can negatively impact the long-term performance of the grout. In Figure 14e, substantial NS agglomeration is observed, attributed to enhanced van der Waals forces at the nanoscale when NS content is excessive. As particle spacing decreases, these forces increase sharply, promoting aggregation. Macroscopically, this leads to reduced flowability, increased viscosity, greater water demand, and ultimately a decrease in compressive strength.

By comparing SEM images at different NS dosage levels, the evolution of the microstructure in cement-based grouting materials can be clearly observed. In the control group without NS addition (G2), CH crystals exhibit coarse plate-like structures with disordered arrangement, accompanied by numerous isolated pores, resulting in poor overall continuity. When the NS content increased to 0.4% (Group N2), the microstructure underwent significant changes: most CH crystals exhibited regular hexagonal plate-like arrangements, while AFt particles distributed uniformly throughout the matrix (Figure 14b). This optimized composite morphology effectively reduced stress concentration between crystals: on one hand, the refinement of CH crystals mitigated local stress risks caused by oversized particles, while the needle-like AFt structure enhances overall connectivity through bridging effects. Its interlaced arrangement with CH crystals further disperses stress concentration zones. Regarding pore structure, microscopic observations of the N2 group (0.4% NS content) reveal the complete disappearance of large pores, replaced by micropores, resulting in reduced total porosity. This pore refinement phenomenon can be attributed to the “particle-gel” composite filling effect of NS particles (NS and C-S-H form an interpenetrating dense structure, i.e., the “particle-gel” composite filling system). Notably, the flowability of this group decreased by only 8% (compared to the control group G2), indicating that the filling effect of NS did not significantly hinder slurry flow.

Overall, the incorporation of nanosilica (NS) greatly enhances the hydration process through its pozzolanic activity, nucleation effect, and micro-filling capabilities. These effects promote the formation of AFt crystals and C-S-H gel, thereby improving the overall strength of the grout. However, excessive NS content can sharply increase the consistency and viscosity of the cement paste, significantly reducing its flowability. This can lead to the entrapment of harmful gases and pore formation during casting, which in turn lowers the material’s density and mechanical properties.

### 3.4. Analysis of Uniaxial Tensile Test

#### 3.4.1. Fine Failure Form Analysis

**Figure 15:**
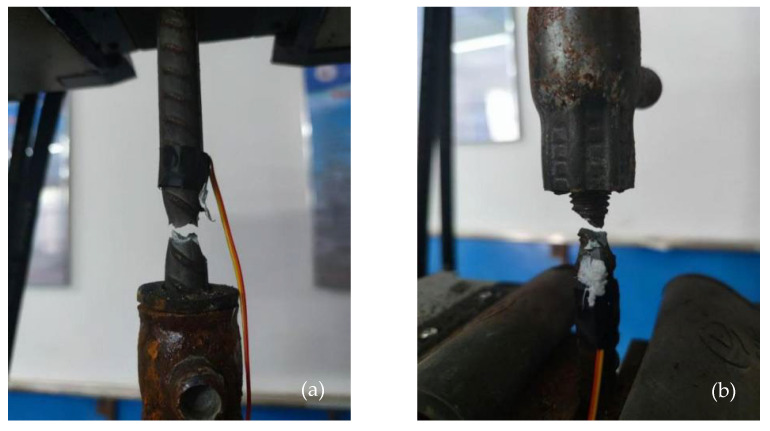

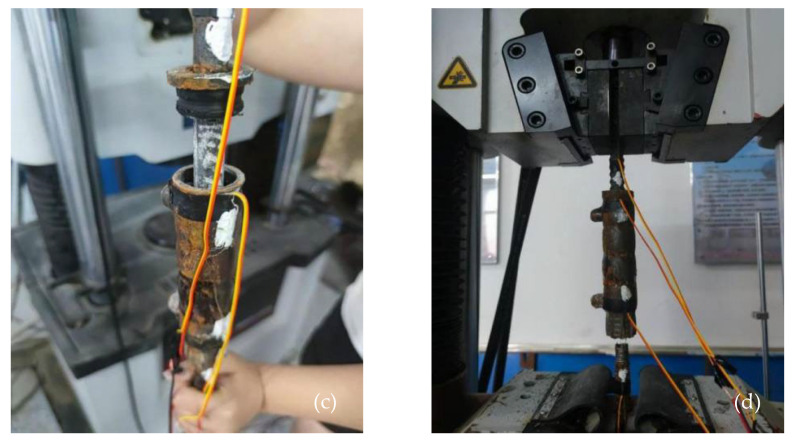
Failure form of sleeve: (**a**) The upper reinforcement is broken; (**b**) Rebar at threaded end is broken; (**c**) Reinforcement pulled out; (**d**) Thread slide is pulled out.

According to the Technical Specification for Application of Grouted Sleeve Connections for Reinforcement (JGJ 355-2015), the tensile strength of the grouted sleeve connection joint shall not be less than the standard tensile strength of the connected reinforcing bar, and failure should occur outside the sleeve, in the reinforcing bar itself. In the uniaxial tensile tests of the grouted sleeve specimens conducted in this study, failure modes included necking fractures at the upper end of the rebar, necking fractures at the threaded end, rebar pullout from the upper end, and rebar pullout due to thread slippage. No rupture of the grouted sleeve itself was observed throughout the tests. Detailed results are shown in Table 10 and Figure 15.

The test results show that all specimens in the control group experienced internal sleeve slippage leading to rebar pullout, with ultimate tensile loads reaching the standard tensile strength of the reinforcing bars. For specimens with a 16 mm rebar diameter, all grout sleeves with nanosilica (NS) additions fractured, with one specimen in group N4 failing due to tensile failure initiated at the rebar thread region. For specimens with an 18 mm rebar diameter, all rebars in group N4 were pulled out from the upper end of the grout sleeve, indicating the poorest compatibility with the sleeve. In contrast, all rebars in group N2 fractured, while groups N1, N3, and N5 each had one specimen that fractured and one that exhibited pullout from the upper end of the sleeve. Regardless of the rebar diameter, specimens exhibiting pullout generally showed larger residual deformations but smaller total elongations.

A comprehensive analysis of residual deformation and total elongation data reveals particular cases in groups N3 and N4. In group N3, the pullout occurred at the threaded end of the half-grouted sleeve, with relatively large residual deformation and smaller total elongation compared to other specimens with 18 mm rebars. This suggests that the pullout from the lower end may be due to a quality issue in individual grout sleeves. In group N4, the rebar fractured at the threaded end within the sleeve, with residual deformation and total elongation values meeting the standard specifications and comparable to those of other groups. Therefore, this type of failure is considered within the normal range of rebar fracture.

Overall, the increase in grout sleeve size partially affects the workability of the solid waste cement-based grout containing nanosilica (NS). Nevertheless, the addition of NS enhances the workability of the solid waste cementitious grout. Among the tested groups, group N2, with an NS content of 0.4%, exhibited the best performance and showed superior compatibility with grout sleeves of both sizes.

#### 3.4.2. Load–Displacement Curve Analysis

**Figure 16:**
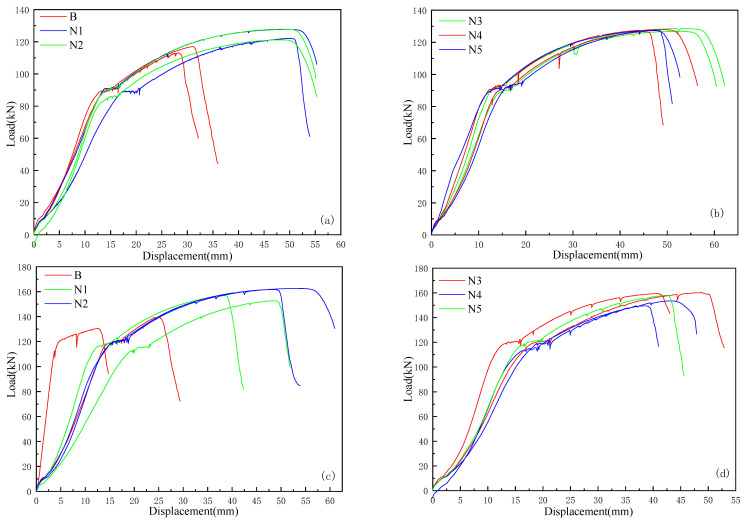
Load displacement curve of Ф16 mm steel bar sleeve: (**a**) B, N1, N2; (**b**) N3, N4, N5; Load displacement curve of Ф18 mm steel bar sleeve; (**c**) B, N1, N2; (**d**) N3, N4, N5.

Load–displacement curves were plotted based on the experimental data. In these figures, curve B represents the half-grouted sleeve group without nanosilica (NS) addition, corresponding to the control group G2 from preliminary tests. Curves N1 through N5 represent half-grouted sleeve groups with NS contents of 0.2%, 0.4%, 0.6%, 0.8%, and 1.0%, respectively. As shown in Figure 16a,b, for grout sleeves with a diameter of 16 mm, the load–displacement curves of groups N1–N5 are generally similar. In contrast, the load for group B declines when the displacement is less than 30 mm, indicating premature load loss due to rebar pullout before reaching the standard tensile strength. Figure 16c,d shows the load–displacement behavior for 18 mm grout sleeves. Due to differing failure modes among the groups, the curves vary considerably; however, their initial ascending trends are nearly identical. Notably, groups N3 and N4 exhibit significant fluctuations during the strengthening phase, likely caused by progressive damage within the grout under loading, which leads to rebar slip inside the sleeve and eventual pullout.

A comprehensive analysis of Figure 16a–d shows that the nanosilica (NS)-modified solid waste grout has better compatibility with steel bars with a diameter of 16 mm. For the grouting sleeve with a diameter of 18 mm, the load–displacement curves exhibit greater fluctuations; however, most groups still reach the ultimate load capacity of the rebar, followed by necking and fracture. The ultimate load increases with the size of both the rebar and the sleeve. Properly designed grout sleeve connections predominantly fail by necking fracture of the reinforcement. The ascending portions of the load–displacement curves for different sleeve sizes are similar, typically including linear elastic, yielding, strain-hardening, and necking stages.

#### 3.4.3. Stress–Strain Curve Analysis

**Figure 17:**
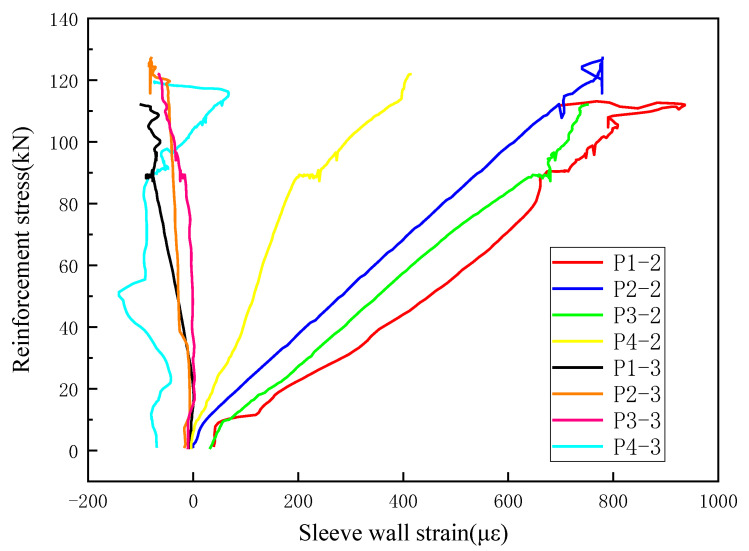
Stress–strain curves of cylinder walls in groups N1–N4.

Analysis of Figure 17 shows that during the pullout tests, the strain gauges on grout sleeves containing nanosilica (NS) exhibit generally consistent trends across all groups. The strain in the sleeve walls remains nearly linear, indicating good integration between the grout sleeve and the grout material, which effectively transfers the tensile stress applied by the reinforcing bars. Once the applied load reaches a certain threshold (corresponding to rebar fracture), the strain curves begin to fluctuate irregularly and unpredictably. Considering the failure modes, this phenomenon is likely caused by rebar fracture occurring at the threaded end in group N4, resulting in relatively smaller strain values recorded by strain gauge No. 2 compared to other groups. Additionally, strain gauge No. 3 in group N4 displays irregular fluctuations from the onset of loading.

B, N1, N2, N3, and N4 represent different grout groups. The numbers 2 and 3 following each group indicate the strain gauge numbers; for example, N1–2 refers to strain gauge No. 2 in group N1.

**Figure 18:**
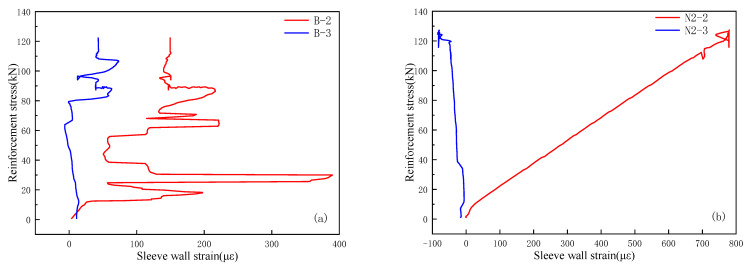
Stress–strain curve of sleeve wall: (**a**) group B; (**b**) group N2.

Figure 18a,b present data from the control group and group N2, respectively. As shown in Figure 18a, the sleeve strain in the control group exhibits irregular fluctuations, with strain gauge No. 2 displaying particularly large variations. This phenomenon is attributed to poor bonding between the grout and the sleeve wall inside the specimen. During progressive loading, the grout gradually debonds from the inner sleeve surface, resulting in persistent fluctuations in the sleeve wall strain. In contrast, Figure 18b shows an approximately linear relationship between sleeve wall strain and rebar stress in group N2, further confirming that the nanosilica (NS)-modified solid waste grout bonds well with the sleeve wall, indicating strong compatibility between the grout and the sleeve.

## 4. Conclusions

In summary, based on the current scope and findings of this research, the study explores the modification of solid waste-based sleeve grouting materials using nanomaterials. Experimental results confirm that specific nanomaterials, such as nano-silica, significantly enhance the performance of grouting materials. However, the current assessment of nanomaterial modification effects primarily focuses on single nanomaterials within specific dosage ranges, without extensive comparative analysis involving multiple nanomaterials. Subsequent sections will detail specific findings regarding performance enhancements in grouting materials. To comprehensively define the positioning of the developed nanomaterial-modified grouting materials within nanotechnology, future research will conduct in-depth comparisons with a broader range of known nanomaterials. This will provide valuable insights for further optimizing material properties and expanding their application potential.

- The addition of nanosilica (NS) reduces the flowability of the grout. Within the range of 0.2% to 1.0% NS content, the early compressive strength of the material is minimally affected; however, it significantly enhances the later-stage compressive strength of the cement-based grout. The optimal NS dosage is 0.4%, at which the 28-day compressive strength reaches a maximum of 105.5 MPa, representing an increase of 37.9% compared to the control group without NS.
- With the incorporation of NS, the hydration products such as ettringite (AFt), hydrated calcium aluminate, and tricalcium silicate in the grouting material increased. A large amount of NS can be adsorbed on the surfaces of silica fume, fly ash, and calcium hydroxide, which accelerates the consumption of calcium hydroxide, promotes the hydration process, and consequently facilitates the formation of hydration products such as AFt and C–S–H gel.
- The ultimate load capacity of grout sleeves increases with the size of both the rebar and the sleeve. Well-performing grout sleeves exhibit similar ascending stages in their load–displacement curves, including linear elastic, yielding, strain-hardening, and necking failure phases. Prior to rebar fracture, the strain in the sleeve wall increases approximately linearly with the increase in rebar stress.
- The solid waste cement-based grout containing nanosilica (NS) demonstrates good compatibility with semi-grout sleeves with a diameter of 16 mm and 18 mm. Among the tested dosages, the grout with 0.4% NS exhibits the best performance and strongest compatibility in pullout tests for both sleeve sizes.

## Figures and Tables

**Table 1:** Performance indicators of ordinary Portland cement.

Specific Surface Area (m^2^·kg^−1^)	Flexural Strength (MPa)		Compressive Strength (MPa)		Setting Time (min)	
	3 d	28 d	3 d	28 d	Initial	Final
380	4.1	7.7	25.6	58.8	100	160

**Table 2:** Particle size distribution of silica fume.

Fineness (nm)	80~160	160~280	≥280
Proportion	28.4%	45.2%	26.4%

**Table 3:** Chemical composition of silica fume and fly ash.

Composition	CaO	SiO_2_	Fe_2_O_3_	MgO	K_2_O	NaO	C
Silica fume	1.86	92.99	0.58	0.26	0.88	0.15	1.05
Fly ash	1.08	58.69	5.89	2.82	0.23	0.28	0.88

**Table 4:** Basic performance indicators of nano silica.

Bulk Density (g·cm^−3^)	Density (g·cm^−3^)	Pureness (%)	Crystal Type
0.12	2.2–2.6	99.9	sphericity

**Table 5:** Performance indicators of quartz sand and iron tailings sand.

Indicator	Bulk Density (g·cm^−3^)	Density (g·cm^−3^)	Porosity (%)	Fineness
quartz sand	1.80	2.65	43.8	2.2
iron tailings sand	1.15	1.49	49.4	2.1

**Table 6:** Mechanical performance indicators of steel bars.

Diameter (mm)	Tensile Strength (MPa)	Yield Strength (MPa)	Tensile to Yield Ratio	Yield Ratio
16	620	460	1.34	1.25
18	625	450	1.38	1.28

**Table 7:** Physical indicators of grouting sleeve.

Sleeve Category	Quality (kg)	Diameter (mm)	Sleeve Length (mm)	Thread Segment Length (mm)
D16	0.58	38.5	174	26
D18	0.79	42	193	29

**Table 8:** Grouting material mix design.

Group	Silica Fume (g)	Fly Ash (g)	Quartz Sand (g)	Iron Tailings Sand (g)	NS Proportion (%)	Cement (g)	Water (g)
G2	135	45	720	180	/	720	288
N1	135	45	720	180	0.2	720	288
N2	135	45	720	180	0.4	720	288
N3	135	45	720	180	0.6	720	288
N4	135	45	720	180	0.8	720	288
N5	135	45	720	180	1.0	720	288

**Table 9:** Requirements for joint type test and inspection.

**Uniaxial Tension**	Percentage total extension at maximum force (%)	Asgt ≥ 6.0
	Residual deformation (mm)	u0 ≤ 0.10 (d ≤ 32)
		u0 ≥ 0.14 (d > 32)

**Table 10:** Half-grouted sleeve uniaxial tensile test results.

Group	Sleeve Size (mm)	Displacement (mm)	Ultimate Tensile Load (kN)	u_0_ (mm)	$A_{sgt}$	Failure Mode
B	16	28.05	113.20	0.08	4.62	pulled out
		31.13	116.92	0.08	4.88	pulled out
	18	12.54	130.48	1.0	4.23	pulled out
		25.14	139.66	0.07	7.21	pulled out
N1	16	49.82	127.65	0.04	10.82	pulled apart
		45.03	121.63	0.05	10.36	pulled apart
	18	48.52	152.67	0.04	12.41	pulled apart
		38.57	156.75	0.08	12.12	pulled out
N2	16	47.04	128.41	0.05	10.87	pulled apart
		49.23	127.83	0.04	10.75	pulled apart
	18	52.34	162.56	0.06	12.69	pulled apart
		48.73	161.68	0.06	12.68	pulled apart
N3	16	52.18	126.83	0.04	10.77	pulled apart
		54.04	128.50	0.05	10.82	pulled apart
	18	40.75	159.43	1.0	8.92	pulled out (threaded end)
		48.78	160.22	0.07	12.32	pulled apart
N4	16	45.58	126.62	0.04	10.82	pulled apart
		48.94	127.91	0.05	10.36	pulled apart (threaded end)
	18	38.55	156.56	0.09	7.63	pulled out
		43.14	153.46	0.09	7.55	pulled out
N5	16	47.29	127.28	0.06	10.89	pulled apart
		47.03	127.53	0.05	10.92	pulled apart
	18	42.61	158.05	0.06	11.87	pulled apart
		19.25	133.45	0.08	5.16	pulled out

## Data Availability

The original contributions presented in the study are included in the article. Further inquiries can be directed to the corresponding author.

## Abbreviations

The following abbreviations are used in this manuscript:

NS	Nanosilica
CH	Calcium hydroxide
SF	Silica fume
FA	Fly ash
SEM	Scanning electron microscopy
XRD	X-ray diffraction
